# Outcomes of a 12-month patient-centred medical home model in improving patient activation and self-management behaviours among primary care patients presenting with chronic diseases in Sydney, Australia: a before-and-after study

**DOI:** 10.1186/s12875-020-01230-w

**Published:** 2020-08-08

**Authors:** James Rufus John, W Kathy Tannous, Amanda Jones

**Affiliations:** 1 Translational Health Research Institute (THRI), Western Sydney University, Locked Bag 1797, Penrith, New South Wales 2751 Australia; 2 Rozetta Institute (Formerly Capital Markets Cooperative Research Centre), Level 4/55, Harrington Street, The Rocks, Sydney, New South Wales 2000 Australia; 3 School of Business, Western Sydney University, Parramatta, NSW 2150 Australia; 4 Sonic Clinical Services, Level 21, 225 George Street, Sydney, New South Wales 2000 Australia

**Keywords:** Patient activation, Self-management, Multimorbidity, Chronic disease, Integrated care, Collaborative care, Chronic care model

## Abstract

**Background:**

Studies report that increased levels of patient activation is associated with increased engagement with the health care system, better adherence to treatment protocols, and improved health outcomes. This study aims to evaluate the outcomes of a 12-month Patient-Centred Medical Home (PCMH) model called ‘WellNet’ on the activation levels of patients with one or more chronic diseases in general practices across Northern Sydney, Australia.

**Methods:**

A total of 636 patients aged 40 years and above with one or more chronic conditions consented to participate in the WellNet program which was delivered across six general practices in Northern Sydney, Australia. The WellNet intervention includes team-based care with general physicians and trained chronic disease management care coordinators collaborating with patients in designing a patient-tailored care plan with improved self-management support and care navigation according to the level of risk and health care needs. The level of patient activation was measured using the validated PAM 13-item scale at baseline and follow-up. A before and after case-series design was employed to determine the adjusted mean differences between baseline and 12-months using repeated measures analysis of covariance (ANCOVA). Additionally, the backward stepwise multivariable regression models were employed to identify significant predictors of activation at follow-up.

**Results:**

Of the 626 patients, 420 reported their PAM scores at follow-up. The mean (SD) baseline PAM score was 57.9 (13.0). The adjusted model showed significant mean difference in PAM scores by increase of 6.5 (95% CI 5.0–8.1; *p*-value< 0.001) after controlling for baseline covariates. The multivariable regression models showed that older age (B = − 0.14; 95% CI -0.28, − 0.01) and private insurance (uninsured patients) (B = − 3.41; 95% CI -6.50, − 0.32) were significantly associated with lower PAM scores at 12 months whereas higher baseline PAM score (B = 0.48; 95% CI 0.37, 0.59) was significantly associated with higher follow-up PAM score.

**Conclusion:**

The WellNet study is the first of its kind in Australia to report on changes in the patient activation levels among patients with one or more chronic diseases. PCMH has the potential to improve patient activation and engagement which can lead to long-term health benefits and sustained self-management behaviours.

## Background

The prevalence of multiple chronic conditions is on the rise which presents a significant burden to healthcare systems in Australia and worldwide [1, 2]. Recent advances in medicine and technology have resulted in increased life expectancy which has contributed to growth of the ageing population, surviving with increased years of disabilities and accumulation of chronic conditions [3, 4]. The Australian National Health Survey (NHS) 2017–18 data shows that chronic disease prevalence increases with age, with 80% of Australians aged 65 years and above having one or more chronic conditions [5]. There is well-documented evidence of multimorbidity associated with increased risk of mortality [6]; reduced functional status and quality of life [7]; and increased health service utilisation [8, 9]. Furthermore, patients presenting with multimorbidity are often recipients of fragmented care, as healthcare systems, including Australia, still remain largely configured to management of single diseases, thereby lacking coordination and continuity of care [10]. Contrarily, there is sound evidence suggesting that collaborative approaches in primary care are associated with effective management of chronic illnesses [11, 12].

Management of multimorbidity requires effective care delivery with emphasis on patient-tailored self-management treatment strategies for better patient outcomes [11]. Patient-centred medical home (PCMH) model enables continuity of care through a comprehensive and coordinated approach and aims to improve self-management behaviours configured to individual needs [13, 14]. This enhanced primary care model is led by a general practitioner (GP) as part of a multidisciplinary team (MDT) of primary care providers, working together with patients, to promote proactive care that is targeted to the level of risk and complexity of patients [13, 15]. There is an increasing evidence on the effectiveness of PCMH models of care, primarily in United States, in improving patient activation and self-management of chronic diseases, leading to better quality of health and health utilisation outcomes [16–18]. However, the feasibility and effectiveness of the PCMH model remain unclear in Australian primary care practices.

In the past decade, there has been a growing advocacy towards patient engagement and self-management of chronic disease/s [15, 19]. Patient activation is defined as a multidimensional construct of one’s readiness and ability to manage their own health as well as proactively engaging in making informed decisions about healthcare [20, 21]. The level of activation and ability to self-manage conditions play an important role in a patient’s overall health and wellbeing, especially for those presenting with multimorbidity [20, 22]. There is a growing body of evidence indicating that patients who are actively engaged with the health care team have improved health-related quality of life and clinical outcomes [23–25]. In addition, patients with high activation levels are often empowered through shared decision-making with their GPs and are reported to have better adherence to the treatment regimens and lower hospital admissions compared to patients with lower activation scores [26, 27].

Understanding an individual’s level of activation enables physicians to provide patient-tailored care according to the severity and complexity of the disease [28]. Although chronically ill patients generally report low activation and have increased health care utilisation [26], their levels of activation can be modified through effective education and training [29]. Despite the growing advocacy towards patient activation and self-management, there is relatively little information about the levels of patient activation among individuals presenting with chronic illnesses in Australia. Therefore, this study aims to evaluate changes in the mean patient activation scores and to investigate significant predictors of patient activation following a 12-month PCMH intervention among individuals presenting with one or more chronic diseases in Sydney, Australia.

## Methods

### WellNet program and study design

Sonic Clinical Services (SCS) designed a 12-month chronic disease management (CDM) program called ‘WellNet’ which aims to provide a GP-led, MDT based care for patients with one or more chronic conditions. This enhanced primary care program is built upon the principles of the PCMH model and is guided by other evidence based, best practice models of clinical care in order to deliver high quality patient-centric care that is configured to individual risk and complexity levels [30].

Patients were recruited between December 2016 and October 2017 using a targeted convenience sampling technique. Targeted convenience sampling is a commonly used non-probability sampling method in clinical research where members of the target population that meet certain practical eligibility criteria are included for the purpose of the study [31]. We used a before-and-after case-series study design to evaluate the outcome of WellNet program in improving patient activation levels among primary care patients enrolled in six general practices in Northern Sydney, Australia. A written informed consent was obtained from the participants who enrolled in the 12-month study. A detailed description of the program design and evaluation are reported elsewhere [30].

### Participants

The Participant, Intervention, Comparator, and Outcome statement (PICO) is briefly summarised in Box 1 (supplementary material). Potentially eligible patients (*N* = 1790) were contacted either through letter invites (*N* = 1431) or GP referrals (*N* = 359) for the initial assessment. Eligibility criteria included patients aged 40 years and above; having one or more chronic condition/s; who had consulted a GP three times in the previous 2 years; and had a Hospital Admission Risk Profile (HARP) score of more than 10. The HARP risk assessment tool determines the likelihood of people with chronic or complex care needs presenting to the hospital for treatment in the following 12 months [32]. In addition, patients with one or more consistently elevated clinical risk factors were also invited to participate through GP referrals. Further details of the patient algorithm, recruitment outcomes, and data collection are reported elsewhere [30].

**Fig. 1:**
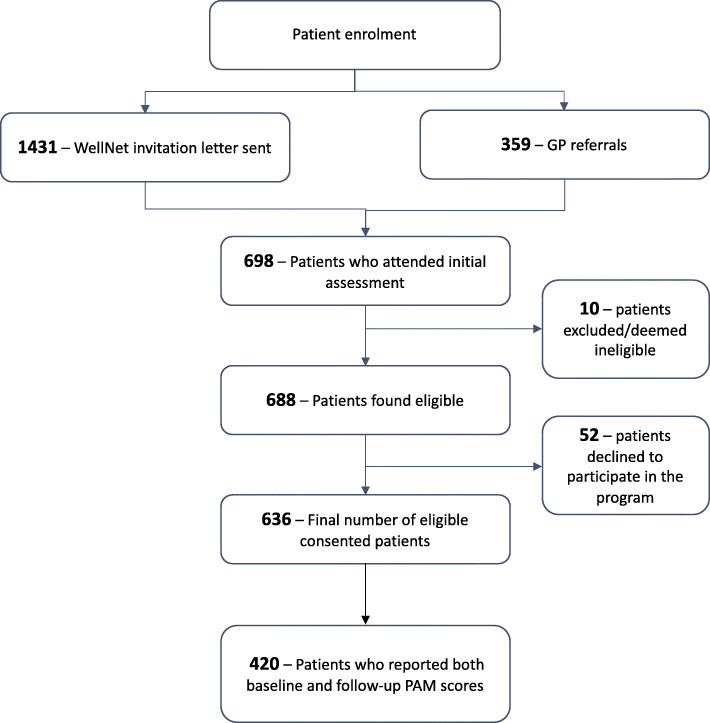
Flowchart of patient enrolment

Of the 1790 invited patients, 698 attended the initial assessment for eligibility and 10 patients were deemed ineligible to participate in the program due to reasons of living in nursing homes and diagnosis of severe cognitive impairment or terminal illness. Out of the eligible 688 patients, 52 declined to participate in the program due to unknown reasons, resulting in 636 patients. Of the 636 consenting participants, 626 reported their baseline PAM score and were included in this study. The flowchart of patient recruitment outcomes is shown in Fig. 1. Although the WellNet study includes a well-matched comparison group based on age, gender, type and number of chronic conditions, self-reported health assessments such as PAM assessments were recorded only among the treatment group. Therefore, the analyses were limited to within-group rather than between-group comparison with standard primary care (comparison group).

### WellNet intervention

**Fig. 2:**
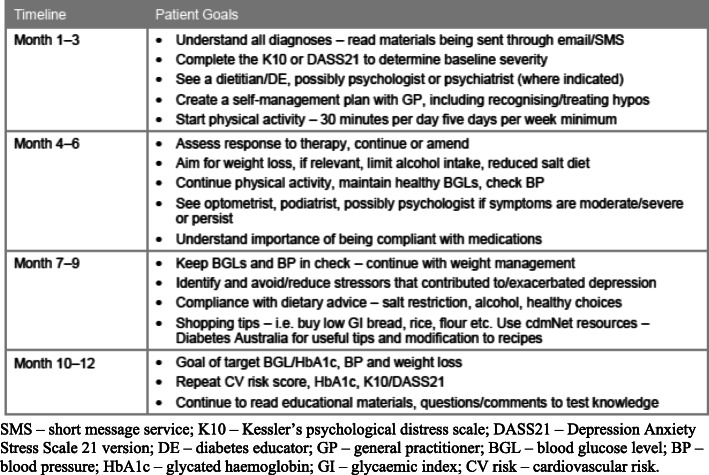
Sample goal chart for patients with type 2 diabetes, hypertension, and depression with timeline. SMS – short message service; K10 – Kessler’s psychological distress scale; DASS21 – Depression Anxiety Stress Scale 21 version; DE – diabetes educator; GP – general practitioner; BGL – blood glucose level; BP – blood pressure; HbA1c – glycated haemoglobin; GI – glycaemic index; CV risk – cardiovascular risk

The 12-month WellNet program is designed to integrate GPs with specially trained chronic disease management (CDM) Care Coordinators (CC) within each of the six participating GP practices [30]. On entry to the program, the team of GPs and CCs coordinate with patients to undertake a range of validated general and disease-specific risk assessments to determine patient’s baseline health status and wellbeing. The information gathered from these assessments is then used to formulate an individualised CDM plan in consultation with the patient. Included in the care plan are patient driven health goals; modifying and training core skills to self-manage symptoms and medications; improving diet and physical activity; and reducing smoking and alcohol consumption [30]. The care plan includes, and is shared with, all relevant members of the care team. Ongoing support to increase knowledge, understanding and maintenance of positive behaviour change; monitoring of progress towards health goals; and assistance to access health and social care are provided through a combination of in-practice and telephone contacts. A sample goal chart with timelines used by the care team to provide individualised care for patients diagnosed with type 2 diabetes, hypertension and depression is shown in Fig. 2.

Furthermore, ongoing patient support is also supplemented through a user-friendly online platform and a mobile application. “GoShare” is an online web-based tool that enables digital sharing of evidence-based patient-relevant education materials. Patients’ access to the materials and understanding of them are regularly monitored and assessed through self-reported surveys. The CCs focus on low adherence to usage or understanding, so that issues can be resolved. Furthermore, patients are also supported with a mobile application, called ‘MediTracker’, which links directly to their clinical records held at the practice, providing access to information such as their current medications, pathology results, diagnoses and immunisation status [30]. This is intended to encourage and empower patients to play an active role in their chronic disease care management.

### Patient activation measure 13 item version

Patient activation was measured with use of the validated PAM-13 item version developed by Hibbard et al. [21]. PAM-13 is a self-reported questionnaire composed of 13 items relating to patients’ beliefs about healthcare, knowledge about their health condition, and confidence in managing health related tasks. Each item has five response options from 0 to 4 such as: (0) ‘not applicable’; (1) ‘strongly disagree’; (2) ‘disagree’; (3) ‘agree’; and (4) ‘strongly agree’. The raw responses range from 13 to 52 which are then transformed through Insignia’s proprietary natural logarithm to a standardised metric ranging from 0 to 100 (0 = lower activation; 100 = highest activation). The scores are classified into four levels of activation: Level 1 (≤47.0) – not believing activation is important; Level 2 (47.1–55.1) – Lacking knowledge or confidence in self-management of health; Level 3 (55.2–67) – Beginning to take action; and Level 4 (≥67.1) – Taking action but require support in maintaining positive behaviour change. Each of these levels provide insights into a range of health-related characteristics, including behaviours and outcomes [28]. In addition, determining baseline scores allow MDT to determine the best approach to engage and educate patients and thus improve self-management behaviour. Studies reporting on validation of PAM scores indicate that the minimal clinically important difference (MCID) is at least a 4-point difference in PAM score in addition to transitioning from lower to higher PAM levels [20, 21]. MCID refers to the smallest change in an outcome score that is considered “important” or “worthwhile” by the practitioner and/or resulting in a change in patient management [33]. Changes in outcomes exceeding this minimal threshold are considered clinically relevant [33].

PAM scores were recorded from the patients at the start and completion (12 months) of the WellNet program. Aligned to the outcome of patient activation, key demographic information of age, gender, type and number of chronic conditions, private health insurance status, and total program contacts were analysed in this study.

### Self-management impact and readiness to change scale of HARP assessment

Item six of the HARP assessment reports on the self-management and readiness to change behaviours which includes several categories: No capacity for self-management; pre-contemplation (not ready for change) and contemplation (considering but unlikely to change); preparation (intending to take action in the immediate future); action (actively changing health behaviours) and maintenance (maintained behaviour for ≥6 months); and relapse. This scale was only used as a supplement to the PAM assessment.

### Study outcomes

The primary outcome of interest for this study was changes in the mean PAM score between baseline and 12-months after controlling for potential baseline covariates such as age, gender, type and number chronic disease diagnosis, insurance status, median visits, and baseline PAM score. Secondary outcomes include: 1) changes in proportion of patients with respect to different levels of PAM and HARP’s self-management impact scale at follow-up; 2) association between PAM levels and self-management impact and readiness to change scale of the HARP risk assessment tool; 3) significant predictors of PAM scores at follow-up.

### Data analysis

Descriptive statistics for continuous variables using mean and standard deviation (SD) and percentages for categorical measures are presented in Table 1. One-way analysis of variance (ANOVA) was conducted to test for significant between-group differences in means between levels of patient activation corresponding to each variable at baseline. Pearson’s correlation coefficient test was also used to determine the association within and between PAM scores and HARP’s self-management impact scale at baseline and follow-up respectively. Additionally, t-tests and chi-square tests were performed to determine any significant difference between patients who completed the program and those who withdrew from the program.

**Table 1:** Patient characteristics by level of patient activation at baseline

Variable	Total	PAM Level 1	PAM Level 2	PAM Level 3	PAM Level 4	Activation score Mean (SD)
All participants	626 (100)	121 (19.1)	236 (37.7)	138 (22.0)	131 (20.9)	57.5 (13.1)
Age in years, Mean (SD)*	68.8 (12.9)	70.4 (12.5)	70.5 (12.9)	66.0 (12.9)	67.0 (12.4)	–
**Median age group**						
*≤ 70 years*	318 (50.8)	60 (18.9)	107 (33.6)	80 (25.2)	71 (22.3)	58.2 (13.3)
*> 70 years*	308 (49.2)	61 (19.8)	129 (41.9)	58 (18.8)	60 (19.5)	56.8 (12.8)
**Gender**						
*Males*	313 (50.0)	62 (19.8)	121 (38.7)	69 (22.0)	61 (19.5)	57.1 (12.9)
*Females*	313 (50.0)	59 (18.8)	115 (36.7)	69 (22.0)	70 (22.4)	58.0 (13.3)
**Diagnosis of chronic condition (grouped)**^a^						
*Circulatory system diseases*	214 (34.2)	44 (20.6)	91 (42.5)	41 (19.2)	38 (17.8)	55.7 (11.4)
*Respiratory diseases*	181 (28.9)	39 (21.5)	64 (35.4)	44 (24.3)	34 (18.8)	57.3 (13.5)
*Diabetes*	307 (49.0)	62 (20.2)	112 (36.5)	71 (23.1)	62 (20.2)	57.1 (12.0)
*Musculoskeletal disorders*	267 (42.7)	58 (21.7)	98 (36.7)	54 (20.2)	57 (21.3)	57.5 (13.5)
*Mental illness*	127 (20.3)	34 (26.8)	48 (37.8)	29 (22.8)	16 (12.6)	54.2 (11.4)
*Cancer*	92 (14.7)	15 (16.3)	35 (38.0)	21 (22.8)	21 (22.8)	57.3 (13.1)
**Number of co-existing conditions, Mean (SD)**	1.9 (0.9)	2.1 (1.0)	1.9 (0.9)	1.9 (0.9)	1.7 (0.9)	–
Insurance status**						
*Private*	398 (68.7)	60 (15.1)	152 (38.2)	89 (22.4)	97 (24.4)	59.0 (13.8)
*Uninsured*	181 (31.3)	50 (27.6)	74 (40.9)	34 (18.8)	23 (12.7)	54.1 (10.4)
**Total program contacts**						
*≤ 11 contacts*	320 (51.1)	64 (20.0)	119 (37.2)	74 (23.1)	63 (19.7)	57.4 (13.4)
*> 11 contacts*	306 (48.9)	57 (18.6)	117 (38.2)	64 (20.9)	68 (22.2)	57.7 (12.8)
**Clinical measures**						
*Systolic Blood Pressure (mmHg), Mean (SD)*	138.8 (19.1)	138.6 (23.1)	138.3 (17.7)	139.4 (19.5)	139.3 (17.3)	–
*Diastolic Blood Pressure (mmHg), Mean (SD)*	75.9 (18.2)	74.3 (19.5)	76.2 (16.8)	76.2 (18.0)	76.4 (19.6)	–
*Body Mass Index Kg/m2, Mean (SD)*	29.9 (7.3)	31.2 (10.1)	29.3 (6.1)	30.1 (6.6)	29.6 (6.5)	–
Glycated Haemoglobin (%), Mean (SD)*	6.8 (1.4)	7.1 (1.3)	6.8 (1.3)	6.9 (1.8)	6.4 (1.1)	–
*High Density Lipoprotein Cholesterol (mmol/L), Mean (SD)*	1.3 (0.4)	1.3 (0.4)	1.3 (0.4)	1.3 (0.4)	1.4 (0.4)	–
Low Density Lipoprotein Cholesterol (mmol/L), Mean (SD)*	2.7 (1.1)	2.6 (1.2)	2.6 (1.0)	2.8 (1.1)	2.9 (1.1)	–
Total Cholesterol (mmol/L), Mean (SD)*	4.8 (1.4)	4.8 (1.6)	4.6 (1.2)	5.0 (1.3)	5.2 (1.4)	–
*Triglyceride (mmol/L), Mean (SD)*	1.6 (1.1)	1.6 (0.9)	1.7 (1.2)	1.7 (1.1)	1.6 (1.3)	–

Data presented as N (%) unless specified otherwise

Variables reported as percentages were tested with chi-square analyses and variables reported as means and standard deviations were tested with ANOVA

^a^Specific chronic diseases were grouped as per the International Statistical Classification of Diseases and Related Health Problems (ICD-10) classification

*p-value< 0.05

**p-value< 0.001

Primary analysis included only those who reported both baseline and follow-up scores. Adjusted mean difference between baseline and follow-up was measured using repeated measures ANCOVA to control for baseline potential covariates such as age, gender, type and number chronic disease diagnosis, insurance status, and median visits. A sensitivity analysis was also conducted to evaluate adjusted differences in PAM scores between baseline and follow-up among patients with two or more chronic conditions.

Backward stepwise multivariable regression models were conducted to determine the predictors of PAM scores at 12-month follow-up. Independent baseline covariates tested against follow-up PAM scores in the univariate analysis included: age, gender, type and number chronic disease diagnosis, insurance status, median visits, and baseline PAM score. Any variable with *p*-value of < 0.2 was then included in the multivariable model. The backward stepwise regression approach was used to reduce and create a final model while simultaneously assessing the fitness of the model in order to avoid dropping of non-significant variables that may affect the model fitness. The final model constitutes variables, which when excluded, cause a prominent deviance change (*p* < 0.05) compared to the corresponding *X*^2^ test statistic on the relevant degrees of freedom.

Internal consistency of pre and post PAM-13 items in this study were evaluated using Cronbach’s alpha. R and SPSS (version 25) statistical software were used to conduct all the analyses. Significance level was set as 0.05 and all statistical tests were two-sided.

## Results

### Baseline patient characteristics and activation levels

The sociodemographic characteristics and chronic disease prevalence of the study sample by baseline PAM levels are presented in Table 1. The mean age of the sample was 69 ± 13 years with equal gender distribution. The study patients had a mean number of 2 ± 1 co-existing chronic condition with diabetes (49%), musculoskeletal disorder (43%), and circulatory system disorders (34%) as the most prevalent of chronic conditions. Of the 420 patients, 62% of patients (*n* = 261) had two or more chronic conditions. In addition, more than two-thirds (69%) of patients had private insurance and almost half (49%) of the patients had more than 11 out of a possible 14 program contacts in the 12-month program. As a priori sample size calculation was not performed, post hoc (retrospective) power analysis showed that the study is sufficiently powered (power = 100%, alpha error = 0.05).

At baseline, no significant difference was observed between patients who completed and those who withdrew from the 12-month program. In addition, results of the one-way ANOVA and chi-square tests showed no significant differences by PAM levels with exception for age, private health insurance (PHI) status, glycated haemoglobin (HbA1c), and total cholesterol. Furthermore, the internal consistency of baseline and follow-up PAM in this study was good with Cronbach’s alpha coefficients of 0.90 and 0.91 respectively.

### Primary outcome

#### Changes in mean PAM scores

Of the 626 patients who reported their baseline PAM, 420 (67%) reported PAM levels at program completion. The mean (SD) PAM score at baseline was 57.9 (13.0). Within-group analysis between baseline and follow-up showed significant improvement in mean PAM scores with a mean difference (unadjusted) of 6.8 (95% CI 5.39 to 8.25, *p*-value< 0.001). After adjusting for potential confounders, the adjusted model showed a significant mean difference of 6.6 (95% CI 5.03 to 8.06, *p*-value< 0.001) (Table 2).

**Table 2:** Repeated measures ANCOVA (main and sensitivity analyses)

Analysis	Unadjusted mean difference (95% CI)	Adjusted mean difference (95% CI)
Overall	6.82 (5.39, 8.25)**	6.55 (5.03, 8.06)**
Sensitivity analysis (patients with ≥2 chronic conditions)	7.71 (5.99, 9.43)**	7.54 (5.72, 9.37)*

*p-value< 0.05

**p-value< 0.001

Additionally, the sensitivity analysis of patients with two or more chronic conditions (*n* = 261) showed that PAM scores (unadjusted) increased from a mean (SD) of 56.3 (12.02) at baseline to 64.1 (15.2) at 12 months. After adjusting for potential covariates, an adjusted mean difference of 7.54 (95% CI 5.72 to 9.37, *p*-value< 0.05) was observed (Table 2).

### Secondary outcomes

#### Changes in PAM levels and HARP’s self-management impact levels at follow-up

Cross-tabulation between baseline and follow-up showed significant differences in PAM levels with 43% of patients transitioning from lower to higher levels whereas only 10% transitioned from higher to lower activation levels (*p*-value< 0.001) (Table 3). More specifically, patients in the WellNet program reported positive change in the PAM levels by significantly increasing from the order of least activated levels (9.2% from Level 1; 24% from Level 2; and 39.2% from Level 3) to the most activated Level 4 at follow-up (*p*-value< 0.001) (Table 3).

**Table 3:** A cross-tabulation table of PAM levels pre- and post-intervention

Follow-up PAM				
Baseline PAM	Level 1 (least activated)	Level 2	Level 3	Level 4 (most activated)
Level 1	14 (18.4)	31 (40.8)	24 (31.6)	7 (9.2)
Level 2	12 (7.8)	63 (40.9)	42 (27.3)	37 (24.0)
Level 3	1 (1.0)	10 (10.3)	48 (49.5)	38 (39.2)
Level 4	0 (0.0)	13 (14.0)	9 (9.7)	71 (76.3)

Data represented as N (%)

In terms of the HARP’s self-management impact levels, 60% (*n* = 165) of patients in the contemplation/ pre-contemplation level at baseline transitioned to the action and maintenance level at follow-up whereas 64% (*n* = 73) of patients in the preparation level at baseline transitioned to the action and maintenance level at follow-up (Table 4). Furthermore, Pearson’s correlation coefficient between PAM and HARP’s self-management levels at both baseline and follow-up was less than 0.39 at *p* < 0.01 significance indicating a weak positive association (Table 5).

**Table 4:** A cross-tabulation table of HARP’s self-management impact levels pre- and post-intervention

	Follow-up levels		
Baseline levels	Contemplation/ pre-contemplation	Preparation	Action and maintenance
**Contemplation/ pre-contemplation**	46 (16.8)	62 (22.7)	165 (60.4)
**Preparation**	18 (15.8)	23 (20.2)	73 (64.0)
**Action and maintenance**	2 (9.5)	3 (14.3)	16 (76.2)

Data represented as N (%)

**Table 5:** Correlation test between PAM levels and HARP’s self-management levels at baseline and follow-up

	Baseline PAM levels	Follow-up PAM levels	Baseline HARP levels	Follow-up HARP levels
**Baseline PAM levels**	1	.493**	.190**	.205**
**Follow-up PAM levels**	.493**	1	.186**	.252**
**Baseline HARP levels**	.190**	.186**	1	.062
**Follow-up HARP levels**	.205**	.252**	.062	1

Pearson’s bivariate correlation coefficients

**p-value< 0.01 (two-tailed test)

#### Predictors of change in PAM scores at follow-up

Results of the multivariable regression analyses showing significant predictors of patient activation levels at follow-up are presented in Table 6. Older age, lack of private health insurance (PHI), and higher baseline PAM score were found to be significant predictors. Increase in patient’s age (B = − 0.14, *p* = 0.043) and uninsured patients (B = − 3.41, *p* = 0.033) were significantly associated with decreased PAM scores at follow-up. Conversely, higher baseline PAM score (B = 0.48, *p* < 0.001) was significantly associated with higher follow-up PAM score (Table 6).

**Table 6:** Predictors of patient activation scores at 12-month follow-up

Predictors	(***N*** = 420)	
	B	***p***-value
Age	−0.14 (−0.28, −0.01)	0.043
Insurance status: Uninsured	−3.41 (−6.50, − 0.32)	0.033
Baseline PAM score	0.48 (0.37, 0.59)	< 0.001

B – unstandardized beta coefficient (slope)

## Discussion

This study evaluates changes in the activation levels and investigates significant predictors of patient activation among individuals presenting with one or more chronic conditions in primary care across Northern Sydney, Australia following a 12-month enhanced primary care model. Primary care is well established as the forefront of care delivery in Australia, however research with use of primary care data is relatively low. Moreover, GP practice activity in Australia shows that the management rate of chronic conditions was 55 per 100 encounters and that 96% of encounters among patients aged 65 years and above had one or more chronic conditions [34]. In view of this, the Australian Medical Association (AMA) has acknowledged the importance of primary care as an ideal setting to facilitate patient-centred care and also in educating patients to effectively self-manage their chronic conditions, which could result in better patient outcomes [15].

The 12-month WellNet intervention resulted in both statistically significant and clinically meaningful improvement in PAM scores with adjusted mean differences in activation score of 6.5 after controlling for potential confounders. There is also evidence showing that each point increase in PAM scores is associated with 2% reduction in hospitalisation and 2% improvement in medication adherence [35]. In the secondary outcome analyses, there was a statistically significant difference observed between baseline and follow-up PAM levels where 43% of study patients experienced transition from a lower level to a higher level of activation post-intervention. The improvement in patient activation and self-management behaviours in patients is consistent across several other studies that have incorporated core principles of the PCMH model [36, 37]. Although this study exceeded the MCID and resulted in individual-level transition from one PAM level to another, the follow-up PAM score of 64.6 did not lead to overall sample-level changes as the follow-up score was still in the Level 3 range of PAM. Therefore, there is still more room for improvement in activation which may not have manifested in the relatively short period of 12 months, given, it could take time and sustained education for patients to build confidence to effectively self-manage their chronic illnesses.

In the multivariable regression analyses, increase in age, PHI, and baseline PAM scores were significant predictors of PAM scores at the 12-month follow-up. Increase in years of age as a significant determinant of reduced activation is consistent with findings of studies by Blakemore et al. and Overbeek et al. [38, 39]. Preliminary qualitative feedback of the WellNet program showed that some elderly patients reported difficulty in engaging with the online GoShare tool and MediTracker mobile application, thereby needing more assistance and coaching to access the electronic educational programs. This could be a plausible explanation as to why elderly patients may have had lower levels of activation at follow-up. Consistent with the above study findings, WellNet group observed a slightly higher mean number of co-existing chronic conditions among patients who were over the median age of 70 years compared to those who were less than or equal to 70 years (2.0 vs 1.8, *p*-value = 0.05).

PHI status was a strong predictor of change in activation levels where uninsured patients were associated with significantly lower activation compared to privately insured patients. This may be due to the reason that patients with PHI coverage may have better access to healthcare in terms of choice of providers and shorter waiting times for treatment compared to those without PHI [40, 41]. Furthermore, this study is also consistent with findings of studies by Chubak and Rijken et al. which have shown that patients who were activated at baseline had improved activation scores over time [42, 43].

In comparison to improvements in the PAM levels, a similar positive trend was observed in the HARP’s self-management impact and readiness to change assessment item. However, the weak positive association between HARP item and PAM levels could possibly be due to the fact that patients would have better perceived and responded to several specific items of the PAM assessment as opposed to attempting to respond in an overly positive manner on the single item of the HARP assessment.

This study has several strengths and limitations. To our knowledge, the WellNet program is the first of its kind in Australia to evaluate the outcomes of a PCMH model in improving levels of activation and self-management among patients with one or more chronic conditions using GP data. In addition, the program’s strengths include large sample size, comprehensive data collection by trained healthcare professionals, and longitudinal measurements rendering determining predictors of change in PAM scores. In terms of the study limitations, the lack of control group means that the possibility of potential bias cannot be excluded, and we cannot be sure that improvement in PAM scores may have occurred anyway without the enhanced PCMH intervention. However, that seems unlikely based on trials conducted with use of control groups which reported similar outcomes [20, 44]. In addition, some key socio-demographic and socio-economic variables such as education status and income were unavailable for assessment reducing the ability to identify other predictors of change in patient activation. Finally, consistent with other originally designed programs, reproducibility of findings is constrained by potential barriers in the form of uniqueness of data and by patient and provider-level determinants [30, 45].

## Conclusion

Patient activation is an important precursor not only for effective self-management of chronic conditions but also to empower patients in actively making decisions concerning their health. The integration of GPs and trained CDM coordinators proves critical for provision of individualised care for patients presenting with one or more chronic conditions. This study demonstrates the outcomes of an enhanced primary care model in improving patient activation and self-management outcomes over 12-months. Patients who participated in the WellNet program achieved both statistically significant and clinically meaningful improvements in PAM scores. Findings of this study emphasises the need to increase support for older and uninsured patients in managing their health and healthcare needs. Future research should seek to evaluate the long-term effects and cost-benefits of increased activation in this cohort. Furthermore, additional research is needed to determine disease-specific interactions on patient activation levels. This will render re-designing the level of care to where it is most needed.

## Supplementary information

**Additional file 1.** Box 1 PICO statement.

## Data Availability

Data contained in the WellNet cohort cannot be made publicly available due to privacy reasons.

## Abbreviations

AMA: Australian Medical Association
ANOVA: Analysis of variance
ANCOVA: Analysis of covariance
CC: Clinical coordinator
CDM: Chronic disease management
CI: Confidence interval
CVD: Cardiovascular disease
FCS: Fully conditional specification
GLM: General linear models
GP: General practitioner
HARP: Hospital Admission Risk Profile
MCID: Minimal clinically important difference
MCMC: Markov Chain Monte Carlo
MDT: Multidisciplinary team
NHS: National Health Survey
PAM: Patient Activation Measure
PCMH: Patient Centred Medical Home
PHI: Private health insurance
PICO: Participant, Intervention, Comparator, and Outcome statement
SCS: Sonic Clinical Services
SD: Standard Deviation
SPSS: Statistical Package for the Social Sciences
US: United States

## References

[1] Garin N, Koyanagi A, Chatterji S, Tyrovolas S, Olaya B, Leonardi M, Lara E, Koskinen S, Tobiasz-Adamczyk B, Ayuso-Mateos JL, Haro JM (2015). Global multimorbidity patterns: a cross-sectional, population-based, multi-country study. J Gerontol.

[2] van Oostrom SH, Gijsen R, Stirbu I, Korevaar JC, Schellevis FG, Picavet HS, Hoeymans N (2016). Time trends in prevalence of chronic diseases and multimorbidity not only due to aging: data from general practices and health surveys. PLoS One.

[3] Lunenfeld B, Stratton P (2013). The clinical consequences of an ageing world and preventive strategies. Best Pract Res Clin Obstet Gynaecol.

[4] Ofori-Asenso R, Chin KL, Curtis AJ, Zomer E, Zoungas S, Liew D (2019). Recent patterns of multimorbidity among older adults in high-income countries. Popula Health Manag.

[5] Australian Bureau of Statistics (2018). National Health Survey: First Results, 2017–18.

[6] Gallacher KL, McQueenie R, Nicholl B, Jani BD, Lee D, Mair FS (2018). Risk factors and mortality associated with multimorbidity in people with stroke or transient ischaemic attack: a study of 8,751 UK biobank participants. J Comorb.

[7] Fortin M, Lapointe L, Hudon C, Vanasse A, Ntetu AL, Maltais D (2004). Multimorbidity and quality of life in primary care: a systematic review. Health Qual Life Outcomes.

[8] Salisbury C, Johnson L, Purdy S, Valderas JM, Montgomery AA (2011). Epidemiology and impact of multimorbidity in primary care: a retrospective cohort study. Br J Gen Pract.

[9] Condelius A, Edberg AK, Jakobsson U, Hallberg IR (2008). Hospital admissions among people 65+ related to multimorbidity, municipal and outpatient care. Arch Gerontol Geriatr.

[10] Barnett K, Mercer SW, Norbury M, Watt G, Wyke S, Guthrie B (2012). Epidemiology of multimorbidity and implications for health care, research, and medical education: a cross-sectional study. Lancet..

[11] Camacho EM, Davies LM, Hann M, Small N, Bower P, Chew-Graham C, Baguely C, Gask L, Dickens CM, Lovell K (2018). Long-term clinical and cost-effectiveness of collaborative care (versus usual care) for people with mental–physical multimorbidity: cluster-randomised trial. Br J Psychiatry.

[12] Coventry P, Lovell K, Dickens C, Bower P, Chew-Graham C, McElvenny D, Hann M, Cherrington A, Garrett C, Gibbons CJ (2015). Integrated primary care for patients with mental and physical multimorbidity: cluster randomised controlled trial of collaborative care for patients with depression comorbid with diabetes or cardiovascular disease. BMJ.

[13] Jackson GL, Powers BJ, Chatterjee R, Bettger JP, Kemper AR, Hasselblad V, Dolor RJ, Irvine RJ, Heidenfelder BL, Kendrick AS (2013). The patient-centered medical home: a systematic review. Ann Intern Med.

[14] Maeng DD, Graf TR, Davis DE, Tomcavage J, Bloom FJ (2012). Can a patient-centered medical home lead to better patient outcomes? The quality implications of Geisinger’s ProvenHealth navigator. Am J Med Qual.

[15] Australian Medical Association (2015). AMA Position Statement on the Medical Home – 2015.

[16] Berk-Clark C, Doucette E, Rottnek F, Manard W, Prada MA, Hughes R, Lawrence T, Schneider FD (2018). Do patient-centered medical homes improve health behaviors, outcomes, and experiences of low-income patients? A systematic review and meta-analysis. Health Serv Res.

[17] Peikes D, Chen A, Schore J, Brown R (2009). Effects of care coordination on hospitalization, quality of care, and health care expenditures among medicare beneficiaries: 15 randomized trials. JAMA..

[18] Reid RJ, Fishman PA, Yu O, Ross TR, Tufano JT, Soman MP, Larson EB (2009). Patient-centered medical home demonstration: a prospective, quasi-experimental, before and after evaluation. Am J Manag Care.

[19] The Royal Australian College of General Practitioners (2016). General practice management of type 2 diabetes: 2016–18.

[20] Hibbard JH, Greene J, Tusler M (2009). Improving the outcomes of disease management by tailoring care to the patient's level of activation. Am J Manag Care.

[21] Hibbard JH, Stockard J, Mahoney ER, Tusler M (2004). Development of the patient activation measure (PAM): conceptualizing and measuring activation in patients and consumers. Health Serv Res.

[22] Deen D, Lu WH, Rothstein D, Santana L, Gold MR (2011). Asking questions: the effect of a brief intervention in community health centers on patient activation. Patient Educ Couns.

[23] Greene J, Hibbard JH (2012). Why does patient activation matter? An examination of the relationships between patient activation and health-related outcomes. J Gen Intern Med.

[24] Reynolds R, Dennis S, Hasan I, Slewa J, Chen W, Tian D, Bobba S, Zwar N (2018). A systematic review of chronic disease management interventions in primary care. BMC Fam Pract.

[25] Havas K, Douglas C, Bonner A (2018). Meeting patients where they are: improving outcomes in early chronic kidney disease with tailored self-management support (the CKD-SMS study). BMC Nephrol.

[26] Kinney RL, Lemon SC, Person SD, Pagoto SL, Saczynski JS (2015). The association between patient activation and medication adherence, hospitalization, and emergency room utilization in patients with chronic illnesses: a systematic review. Patient Educ Couns.

[27] Magnezi R, Glasser S, Shalev H, Sheiber A, Reuveni H (2014). Patient activation, depression and quality of life. Patient Educ Couns.

[28] Hibbard JH, Mahoney ER, Stock R, Tusler M (2007). Do increases in patient activation result in improved self-management behaviors?. Health Serv Res.

[29] Frosch DL, Rincon D, Ochoa S, Mangione CM (2010). Activating seniors to improve chronic disease care: results from a pilot intervention study. J Am Geriatr Soc.

[30] John JR, Jones A, Neville AM, Ghassempour S, Girosi F, Tannous WK (2020). Cohort profile: effectiveness of a 12-month patient-Centred medical home model versus standard care for chronic disease management among primary care patients in Sydney, Australia. Int J Environ Res Public Health.

[31] Sedgwick P (2013). Convenience sampling. BMJ..

[32] Sager MA, Rudberg MA, Jalaluddin M, Franke T, Inouye SK, Landefeld CS, Siebens H, Winograd CH (1996). Hospital admission risk profile (HARP): identifying older patients at risk for functional decline following acute medical illness and hospitalization. J Am Geriatr Soc.

[33] Cook CE (2008). Clinimetrics corner: the minimal clinically important change score (MCID): a necessary pretense. J Man Manip Ther.

[34] Britt H, Miller GC, Henderson J, Bayram C, Harrison C, Valenti L, Wong C, Gordon J, Pollack AJ, Pan Y. General practice activity in Australia 2014–15. Sydney: Sydney University Press; 2015.

[35] Hibbard JH, Greene J (2013). What the evidence shows about patient activation: better health outcomes and care experiences; fewer data on costs. Health Aff.

[36] Hibbard JH, Tusler M (2007). Assessing activation stage and employing a “next steps” approach to supporting patient self-management. J Ambul Care Manag.

[37] Fowles JB, Terry P, Xi M, Hibbard J, Bloom CT, Harvey L (2009). Measuring self-management of patients’ and employees’ health: further validation of the patient activation measure (PAM) based on its relation to employee characteristics. Patient Educ Couns.

[38] Blakemore A, Hann M, Howells K, Panagioti M, Sidaway M, Reeves D, Bower P (2016). Patient activation in older people with long-term conditions and multimorbidity: correlates and change in a cohort study in the United Kingdom. BMC Health Serv Res.

[39] Overbeek A, Rietjens JA, Jabbarian LJ, Severijnen J, Swart SJ, van der Heide A, Korfage IJ (2018). Low patient activation levels in frail older adults: a cross-sectional study. BMC Geriatr.

[40] Duckett SJ (2005). Private care and public waiting. Aust Health Rev.

[41] Giacovelli JK, Egorova N, Nowygrod R, Gelijns A, Kent KC, Morrissey NJ (2008). Insurance status predicts access to care and outcomes of vascular disease. J Vasc Surg.

[42] Chubak J, Anderson ML, Saunders KW, Hubbard RA, Tuzzio L, Liss DT, Morales LS, Reid RS (2012). Predictors of 1-year change in patient activation in older adults with diabetes mellitus and heart disease. J Am Geriatr Soc.

[43] Rijken M, Heijmans M, Jansen D, Rademakers J (2014). Developments in patient activation of people with chronic illness and the impact of changes in self-reported health: results of a nationwide longitudinal study in the Netherlands. Patient Educ Couns.

[44] Lara-Cabrera ML, Salvesen Ø, Nesset MB, De las Cuevas C, Iversen VC, Gråwe RW (2016). The effect of a brief educational programme added to mental health treatment to improve patient activation: a randomized controlled trial in community mental health centres. Patient Educ Couns.

[45] John JR, Tannous WK, Jones A (2020). Effectiveness of a patient-centered medical home model of primary care versus standard care on blood pressure outcomes among hypertensive patients. Hypertens Res.

