# Supplementary material for: Outcomes of a 12-month patient-centred medical home model in improving patient activation and self-management behaviours among primary care patients presenting with chronic diseases in Sydney, Australia: a before-and-after study
Source: BMC Fam Pract. 2020 Aug 8;21:158. doi: 10.1186/s12875-020-01230-w (PMC7414685; doi:10.1186/s12875-020-01230-w)
Supplement: Supplementary file 1 — Additional file 1. Box 1 PICO statement. [file 12875_2020_1230_MOESM1_ESM.docx]

**Box 1. PICO statement**

- **Participants** – Patients aged 40 years and above; one or more chronic conditions/ one or more elevated clinical risk factors; at least 3 or more GP visits in the last 2 years; HARP* score > 10.
- **Intervention** – 12-month enhanced primary care program called ‘WellNet’ based on patient-centred medical home model which involves team-based care, patient-tailored chronic disease self-management support, health coaching and education, care coordination, shared decision making, and regular review.
- **Comparator** – PAM scores were not recorded for the comparison group.
- **Outcomes** – **Primary:** Changes in PAM scores and PAM levels of activation; **Secondary**: 1) Changes in proportion of patients with respect to different levels of PAM and HARP’s self-management impact scale at follow-up; 2) association between PAM levels and self-management impact and readiness to change scale of the HARP risk assessment tool; and 3) significant predictors of PAM scores at follow-up.

*HARP risk assessment tool determines the risk of people with chronic or complex care needs presenting to hospital for treatment in the following 12 months.
